# Factors influencing hematoma expansion in delayed brain CT scans of patients with traumatic epidural hematoma

**Published:** 2024-07

**Authors:** Iran Chanideh, Mohammad Reza Akrami, Seyed Erfan Farsian, Maasoumeh Abbasi, Masoud Ghadiri, Tahereh Mohammadi Majd, Tayebeh Najafi, Saeed Gharooee Ahangar

**Affiliations:** ^*a*^ Clinical Research Development Center, Taleqani and Imam Ali Hospital, Kermanshah University of Medical Sciences, Kermanshah, Iran.; ^*b*^ Student Research Committee, Kermanshah University of Medical Sciences, Kermanshah, Iran.; ^*c*^ Department of Research and Technology, Kermanshah University of Medical Sciences, Kermanshah, Iran.

**Keywords:** Brain Trauma, Epidural Hematoma, Brain Hemorrhage, Extradural, Brain CT scan

## Abstract

**Background:**

Epidural hematoma (EDH) is a type of intracranial hematoma commonly observed in trauma patients. This research aims to evaluate the factors contributing to the expansion of traumatic epidural hematoma (EDH).

**Methods:**

This retrospective cohort study examined traumatic patients with EDH admitted to Taleqani Hospital, a prominent Western Trauma Center in Iran, from 2018 to 2023. Patients underwent an initial CT scan, and non-surgical patients received a delayed CT scan approximately 5 hours after the initial scan. Data analysis was performed using SPSS version 25 software.

**Results:**

The study included 274 brain trauma patients with epidural hematoma. Among these patients, 142 (51.8%) did not undergo surgery, and 121 (85.2%) of the non-surgical patients were male. Motor vehicle accidents (MVAs) were the primary cause of EDH in 127 (46.4%) patients. The mean (±standard deviation) initial hematoma size was 8.86 (±9.71), and the mean (±standard deviation) delayed CT scan size was 8.12 (±8.10). In crude linear regression Two variables, namely Hematoma volume in the initial CT scan (P less than 0.001) and Mixed Density (P=0.007), were found to significantly impact the increase in hematoma size in delayed CT scans of non-surgical patients. But in the adjusted linear regression model, only the Hematoma volume in primary CT scan, was significant (P less 0.001). The R Square values were 0.72.

**Conclusions:**

The identification of key variables influencing hematoma volume in delayed CT scans has the potential to guide more effective interventions, thereby improving patient outcomes and reducing trauma-related mortality.

## Introduction

An epidural hematoma (EDH) is a type of intracranial hemorrhage that occurs in the potential space between the outer layer of the dura mater and the inner table of the skull. It represents approximately 2.7% to 4% of Traumatic Brain Injuries (TBIs) and is often associated with concomitant skull fractures.^1,2^ This condition can be categorized as acute, subacute, or chronic based on its chronicity. Acute traumatic EDHs can appear immediately after trauma or within a few hours (early EDH), or may only become apparent in subse-quent brain CT scans, leading to deterioration in the patient's mental status.^3-7^

The incidence of delayed EDH varies, ranging from 9% to 23% across different papulations.^8-10^ While small EDHs generally have favorable prognoses, significant expansion leading to compression of vital brain structure can result in acute neurologic deficits and even fatality. Timely surgical evacuation of expanding hematomas can be life-saving.^11-13^

The mass effect caused by enlarged hematoma can lead to increased intracranial pressure and uncal herniation, which may have devastating consequences. In such cases, immediate evacuation is the sole treatment option to prevent disability and death. Therefore, sound decision-making for early intervention before the expansion of hematoma is critically important.^14^

Understanding the factors predicting EDH expansion is crucial for patient proper management. However, few global studies have been conducted on this issue to date.^15-17^ Given the high incidence of accidents and injuries in Iran and the substantial involvement of the country's young population in such incidents, including their consequences such as epidural hematoma,^18-21^ investigating predictive factors is of utmost importance for enhancing patient management. Therefore, this study was conducted to explore predictive factors of hematoma expansion in trauma patients referred to a trauma center in Iran. 

## Methods 

In this retrospective cohort study, all patients with traumatic epidural injuries who were referred to the Kermanshah Trauma Center from 2018 to 2023 were included using a census method. Upon arrival, patients received primary health care, a medical history was obtained, and a physical examination was conducted. The patient's level of consciousness was assessed using the Glasgow Coma Scale (GCS) by a neurosurgeon at admission. Subsequently, the patients underwent an initial Computed Tomography (CT) scan in the radiology department. A follow-up CT scan was performed approximately 5 hours after the initial scan, and the patients were reassessed. The decision for surgery or medical treatment was made by a medical team led by a neurosurgeon. Patients were then followed up from this point until either discharge or death, and all information was documented. The same CT scan equipment and facility were used for all patients in this study. Additionally, the CT scan results were interpreted by an experienced radiologist using specific criteria for all patients.

**Inclusion / Exclusion Criteria**

Inclusion criteria included clinical confirmation of epidural hematoma by an expert neurosurgeon, while exclusion criteria comprised follow-up treatment in other centers, coagulation disorders, taking anticoagulant agents, and insufficient data in the patient's medical record.

**Variables**

To collect data, a checklist was used. The demographic section included variables such as age (in years) and sex (male/female). Clinical information comprised variables such as hematoma volume in the initial CT scan, GCS (mild/medium/severe), type of trauma (falling/assault/MVA/other), hematoma location (frontal/occipital/temporal/parietal), fractures (no/yes), petrous temporal bone fracture (no/yes), mixed density (no/yes), air density (no/yes), brain contusion (no/yes), subarachnoid hemorrhage (no/yes), EDH or SDH (no/yes), edema (no/yes), and fractures over major cerebral venous sinuses (no/yes). Traditionally, GCS scores from 14-15 indicate mild TBI, scores from 9-13 indicate moderate TBI, and scores from 3-8 indicate severe TBI.

**Statistical Analysis**

The quantitative and categorical variables were presented as mean (± standard deviation) and frequency (%), respectively. To examine the relationship between independent variables and hematoma volume in the secondary CT scan (dependent variable), Crude linear regression and adjusted linear regression method were conducted. A two-sided P value <0.05 was considered statistically significant. The analyses were carried out using SPSS 25 software. 

## Results

In a recent study, 274 patients with epidural hematoma resulting from trauma, ranging in age from 1 to 86 years, were examined. The majority of the patients, 86.9%, were male. Surgery was performed on 48.2% of the patients, and a follow-up CT scan was conducted for 51.8% of the patients five hours after the initial scan. The average age of all patients was 32.97 (±18.81) years, with non-surgical patients averaging 32.83 (±17.50) years. The petrous temporal bone fracture was observed in 8.5% of the patients, while 19.7% had mixed density in the hematoma and 23.2% had air density. Only 2.8% of patients experienced major cerebral venous sinus fracture, and the majority of brain damage, 31.7%, was located in the frontal area. The mean of hematoma size in non-surgical patients was 8.86 in the initial CT scan and 8.12 in the follow-up CT scan (Table 1 ). In crude linear regression a positive and significant correlation was found between the hematoma volume in the primary CT scan and the hematoma volume in the secondary CT scan among non- surgical patients (P<0.001). Furthermore, a significant association was observed between mixed density and the hematoma size in the delayed CT scan (P=0.007). But in the adjusted linear regression model, only the Hematoma volume in primary CT scan, was significant (P<0.001). The R Square values were 0.72. (Table 2)

**Table 1:** Baseline characteristics of the patients with epidural hematoma according to surgical status.

Variables	Subgroups	Total(n=274) N (%)	Surgery patients (n=132) N (%)	Non- surgical patients (n=142) N (%)	P-value*
**Hematoma volume in primary CT scan**	Mean (±SD)	(36.89±)28.17	48.95(±43.54)	8.86(±9.71)	P<0.001
**Age**	Mean (±SD)	(±18.81)32.97	33.10(±20.01)	(17.50±)32.83	0.90
**Sex**	Female	36(13.1)	15(11.4)	(14.8)21	0.65
	Male	238(86.9)	117(88.6)	(85.2)121	
**GCS**	Mild	177(64.6)	64(48.5)	(79.6)113	0.40
	Moderate	40(14.6)	22(16.7)	(12.7)18	
	Severe	57(20.8)	46(34.8)	(7.7)11	
**Type of Trauma**	falling	101(36.9)	40(30.3)	(43)61	0.01
	assault	30(10.9)	10(7.6)	(14.1)20	
	MVAs	127(46.4)	74(56.1)	(37.3)53	
	Other	16(5.8)	8(6.1)	(5.6)8	
**Hematoma Location **	frontal	91(33.2)	46(34.8)	45(31.7)	0.54
	occipital	16(5.8)	6(4.5)	10(7)	
	temporal	59(21.5)	25(18.9)	34(23.9)	
	parietal	50(18.2)	22(16.7)	28(19.7)	
	multiple	58(21.2)	33(25)	25(17.6)	
**Fracture**	No	78(28.5)	31(23.5)	47(33.1)	0.07
	Yes	196(71.5)	101(76.5)	95(66.9)	
**Petrous Temporal Bone Fracture**	No	245(89.4)	115(87.1)	130(91.5)	0.23
	Yes	29(10.6)	17(12.9)	12(8.5)	
**Mixed Density**	No	170(62)	56(42.4)	114(80.3)	P<0.001
	Yes	104(38)	76(57.6)	28(19.7)	
**Air Density**	No	182(66.4)	73(55.3)	109(76.8)	P<0.001
	Yes	92(33.6)	59(44.7)	33(23.2)	
**Brain Contusion**	No	207(75.5)	95(72)	112(78.9)	0.18
	Yes	67(24.5)	37(28)	30(21.1)	
**Subarachnoid Hemorrhage**	No	213(77.7)	97(73.5)	116(81.7)	0.10
	Yes	61(22.3)	35(26.5)	26(18.3)	
**EDH or SDH**	No	239(87.2)	114(86.4)	125(88)	0.68
	Yes	35(12.8)	18(13.6)	17(12)	
**Edema**	No	116(42.3)	35(26.5)	81(57)	P<0.001
	Yes	158(57.7)	97(73.5)	61(43)	
**Major Cerebral Venous Sinuses Fracture**	No	259(94.5)	121(91.7)	138(97.2)	0.04
	Yes	15(5.5)	11(8.3)	4(2.8)	

**Table 2:** Crude and Adjusted linear regression analyzes in non- surgical patients

Variables	Subgroups	Crude linear regression			Adjusted linear regression		
		Standardized Coefficients Beta	CI (%95) §	P-value*	Standardized Coefficients Beta	CI (%95) §	P-value*
**Hematoma volume in primary CT scan**	-	0.84	0.76-0.94	P<0.001	0.84	0.75-0.94	P<0.001
**Age**	-	0.007	-0.008-0.009	0.93	-	-	-
**GCS**	-	-0.04	-0.03-0.02	0.58	-	-	-
**Sex**	Male	-0.01	-0.50-0.43	0.87	-	-	-
	Female				-	-	-
**Type of Trauma**	Falling (Yes/No)	0.01	-0.30-0.35	0.72	-	-	-
	Assault (Yes/No)	-0.08	-0.69-0.25	0.35	-	-	-
	MVA(Yes/No)	0.01	-0.31-0.35	0.88	-	-	-
	Other (Yes/No)	-0.06	-0.41-0.95	0.43	-	-	-
**Hematoma Location**	Frontal (Yes/No)	-0.01	-0.43-0.37	0.88	-	-	-
	Occipital (Yes/No)	0.02	-0.74-0.97	0.79	-	-	-
	Temporal (Yes/No)	0.06	-0.28-0.62	0.46	-	-	-
	Parietal (Yes/No)	0.03	-0.41-0.62	0.68	-	-	-
**Fracture**	No	0.02	-0.30-0.38	0.81	-	-	-
	Yes				-	-	-
**Petrous Temporal Bone Fracture**	No	-0.01	-0.64-0.54	0.86	-	-	-
	Yes				-	-	-
**Mixed Density**	No	0.23	0.15-0.94	0.007	0.03	-0.14-0.30	0.46
	Yes						
**Air Density**	No	0.09	-0.17-0.60	0.28	-	-	-
	Yes				-	-	-
**Brain Contusion**	No	0.05	-0.27-0.50	0.56	-	-	-
	Yes				-	-	-
**Subarachnoid Hemorrhage**	No	-0.03	-0.50-0.32	0.67	-	-	-
	Yes				-	-	-
**EDH or SDH**	No	-0.11	-0.79-0.17	0.20	-	-	-
	Yes				-	-	-
**Edema**	No	0.08	-0.17-0.48	0.34	-	-	-
	Yes				-	-	-
**Major Cerebral Venous Sinuses Fracture**	No	0.05	-0.65-1.25	0.54	-	-	-
	Yes				-	-	-

R Square = 00.72, Adjusted R Square =0.71 Note. P-value*<0.05: Significant, §=Confidence Interval

## Discussion

The purpose of this retrospective cohort study was to identify the factors that influence hematoma expansion in trauma patients with epidural hematoma at the trauma center of Kermanshah province. The study found association between hematoma expansion on delayed brain CT-scan and the variables of primary hematoma volume and mixed density of hematoma.

The study revealed that there was a significant difference in hematoma volume between the primary and delayed CT-scans. Patients with a larger primary hematoma volume were more likely to experience an increase in delayed hematoma volume. This finding aligns with a study by Shobeiri ^22^ which also showed a link between larger primary hematoma volume and increased secondary hematoma volume, though the relationship was not deemed significant. Another study by Joy and colleagues indicated that a hematoma volume of over 80 ml resulted in over 90% patient mortality.

In a prospective study by Das et al.^23^ involving 1,978 operated EDH patients, it was found that a hematoma volume ≥ 60 mL led to poor outcomes in 36% of cases. Conversely, cases with volumes of 21-40 mL and 41-60 mL had good surgical outcomes (96% and 92% respectively). Similarly, Rehman &Karachi^24^ demonstrated that larger hematomas were associated with increased morbidity and mortality.

Based on the findings of this study and previous research, it can be concluded that larger primary hematoma volumes are more likely to lead to increased hematoma in delayed CT-scans or worse outcomes such as death. Among the clinical variables examined, the size of the primary hematoma may provide more valuable information from a clinical perspective and is the most relevant parameter in terms of prognosis. 

In this study and in crude linear regression a significant relationship was found between mixed density and the expansion of hematoma volume, while no relationship was found between air density and hematoma expansion. Various studies have discussed the relationship between air density, mixed density, and the expansion of hematoma volume. For example, Kopacz et al.'s study^25^ found that over 50% of patients with air density and about 50% of those with mixed density experienced a significant increase (over 25%) in the length, height, and volume of the hematoma.^25^

Most of the time, hematoma expansion follows a benign clinical course, provided that prompt and appropriate diagnosis and treatment are carried out, especially if no additional intracranial injuries, including brain contusion, are present. However, the potential intracranial conditions resulting from the type and severity of the trauma are diverse in extent and location, and can exacerbate the consequences of the disease.

Despite the majority of the traumatic brain injury cases involving men in this study, there was no significant relationship between gender and delayed hematoma size increase. Similarly, in Kang et al.'s study,despite a higher number of traumatic cases in the male population, gender did not significantly affect the increase in hematoma volume.^16^ In Talon et al.'s study, where 80% of trauma cases occurred in men, gender did not significantly affect the outcome among traumatic epidural and subdural patients.^26^ It can be concluded that the higher ratio of trauma cases in men may indicate the severity of trauma resulting from exposure to riskier jobs and cultural factors (such as non-adherence to safety guidelines and driving regulations). However, based on the results of various studies, it appears that gender is not a significant factor in the increase of delayed hematoma; nevertheless, further research is recommended. In our study, there was no significant association between age and hematoma volume in the delayed CT scan. Also, Kang et al. ,^16^ showed that age did not have a significant effect on the worsening of the patient's outcome. In contrast to this study, Cepeda et al.^27^ demonstrated a positive and significant relationship between age and traumatic intracerebral hemorrhage (TICH). They stated that age-related structural weaknesses of the capillaries may be an explanation for TICH expansion. Probably this finding can be extrapolated to epidural hematoma. The difference in the results can be attributed to the difference in the study population. Thus, it is not possible to discuss definitively the relationship between the age and EDH expansion. Conducting a meta-analysis study regarding the effects of age can provide more accurate and reliable information.

The analysis did not reveal a significant correlation between GCS level and hematoma size in delayed CT scans. However, Wang et al.'s study^7^ identified GCS as a predictive factor for mortality, severe disability, and hematoma expansion in ICH patients. This finding may potentially be extrapolated to epidural hematoma cases. Al-Ahmadi et al.^28^ demonstrated that patients with normal or near-normal GCS scores were markedly less prone to increased hemorrhage volume and cerebral edema, with patients experiencing up to a three-fold heightened risk of hematoma expansion as their GCS score declined. In our study, GCS did not exhibit predictive capabilities regarding hematoma expansion, possibly due to the immediate transfer of patients with low GCS scores to the operating room. Although EDHs are relatively rare, occurring in less than 1% of head injury patients, they are crucial in the assessment of moderate to severe head injuries.^29^ Importantly, the level of consciousness at admission plays a pivotal role in guiding initial management decisions and should not be disregarded.

Numerous factors contribute to the expansion of contusions and hematoma volume following traumatic brain injury (TBI). Comprehending the impact of these variables on hematoma volume can potentially inform strategies for patient enhancement. The intricate interactions among a patient's observable, inherent, and mutable characteristics can render the combination of factors ambiguous and make prognosticating patient recovery challenging.^30^ Consequently, the evaluation of additional factors may serve to predict outcomes. Early identification of the determinants influencing hematoma volume prediction, coupled with timely, effective intervention, can substantially mitigate adverse consequences.

## Conclusion

Early diagnosis of delayed epidural hematoma and the influencing factors is imperative for facilitating prompt intervention and mitigating severe consequences. Hence, it is recommended that healthcare practitioners are equipped to accurately identify and evaluate the aforementioned risk factors, particularly in patients with a significant primary hematoma and those displaying mixed-density within the hematoma. For this specific patient cohort, a secondary CT scan should be expeditiously conducted subsequent to the initial assessment. Subsequently, the neurosurgery team should base treatment continuation or patient discharge decisions on the findings of the secondary CT scan.
